# Chronic Oxytocin Administration as a Treatment Against Impaired Leptin Signaling or Leptin Resistance in Obesity

**DOI:** 10.3389/fendo.2015.00119

**Published:** 2015-08-05

**Authors:** Jordi Altirriba, Anne-Laure Poher, Françoise Rohner-Jeanrenaud

**Affiliations:** ^1^Laboratory of Metabolism, Department of Internal Medicine Specialties, Faculty of Medicine, University of Geneva, Geneva, Switzerland

**Keywords:** oxytocin, leptin, adipocyte, obesity, diabetes

## Abstract

This review summarizes the existing literature on the effects of oxytocin administration in the treatment of obesity in different animal models and in humans, focusing on the central control of food intake, the oxytocin effects on adipose tissue, and the relationships between oxytocin and leptin. Oxytocin is a hypothalamic nonapeptide synthesized mainly in the paraventricular and supraoptic nuclei projecting to the pituitary, where it reaches the peripheral circulation, as well as to other brain regions. Moreover, leptin modulates oxytocin levels and activates oxytocin neurons in the hypothalamic paraventricular nucleus, which innervates the nucleus of the solitary tract, partly responsible for the brain-elicited oxytocin effects. Taking into account that oxytocin is located downstream leptin, it was hypothesized that oxytocin treatment would be effective in decreasing body weight in leptin-resistant DIO animals, as well as in those with leptin or with leptin receptor deficiency. Several groups have demonstrated that in such animal models (rats, mice, and rhesus monkeys), central or peripheral oxytocin administration decreases body weight, mainly due to a decrease in fat mass, demonstrating that an oxytocin treatment is able to partly overcome leptin deficiency or resistance. Moreover, a pilot clinical study demonstrated the efficiency of oxytocin in the treatment of obesity in human subjects, confirming the results obtained in the different animal models. Larger multicenter studies are now needed to determine whether the beneficial effects of oxytocin treatment can apply not only to obese but also to type 2 diabetic patients. These studies should also shed some light on the molecular mechanisms of oxytocin action in humans.

## 1 Introduction

According to the World Health Organization, 2.8 million people die worldwide due to overweight or obesity, which represents one of the five leading global risks for mortality (1). Aging is another situation that contributes to the development of overweight or obesity. Indeed, it is well known that the body mass index tends to increase throughout adulthood, to level off from the age of 50 onward (2). Moreover, the presence of overweight or obesity in young and middle age was reported to result in adverse consequences for health care costs in older age (3).

Overall, the key component of the obesity epidemic is long-term dysregulation of energy balance comprising increased energy intake and/or reduced energy expenditure. Despite active research and impressive improvements in the understanding of the regulation of energy balance, there are only a very limited number of drugs that can be used for the efficient treatment of obesity and its comorbidities. Given the well-known state of leptin resistance, present in the vast majority of overweight/obese subjects, together with the fact that leptin exerts a whole array of beneficial effects on body weight homeostasis, one of the common approaches is to identify leptin targets that are able to mimic leptin’s effects, thereby bypassing leptin resistance. Along this line, oxytocin was recently considered as a potential interesting candidate, although it was historically recognized for its role in parturition and lactation (4). The aim of this review is to summarize the existing literature demonstrating the effects of oxytocin on the feeding behavior and peripheral metabolism, as well as to describe the interactions between oxytocin and the leptin signaling pathway.

Virtually, all vertebrate species were found to have an oxytocin-like nonapeptide that supports reproductive functions (5, 6). This impressive evolutionary conservation illustrates the significance of oxytocin in the survival of species. In mammals, oxytocin is synthesized mainly in the central nervous system, within both the magnocellular and parvocellular neurons of the paraventricular nucleus (PVN), as well as by the magnocellular neurons of the supraoptic nucleus (SON) in the hypothalamus. Unlike magnocellular oxytocin neurons, which project to the neurohypophysis (posterior pituitary), where they secrete oxytocin into the circulation, parvocellular oxytocin neurons of the PVN project centrally to various brain areas, including the arcuate nucleus of the hypothalamus (ARC) (7), the ventral tegmental area, the nucleus of the solitary tract (NTS), and the spinal cord (5, 6). Oxytocin release also occurs locally in the SON and PVN via somatodendritics. Several peripheral organs, such as the ovary, uterus, placenta, testis, thymus, kidney, heart, blood vessels, skin, and the gastrointestinal (GI) tract also synthesize oxytocin, although to a lesser extent than hypothalamic neurons [for reviews, see Ref. (5, 6, 8)]. To date, only one oxytocin receptor (OXTR) type has been identified. Its wide distribution within the brain (e.g., basal ganglia, hypothalamus, nuclei of the hindbrain), as well as in various peripheral tissues (e.g., heart, thymus, pancreas, adipocytes, and GI tract) (5) is in keeping with its large variety of physiological actions [for review, see Ref. (5)].

In 1989, Arletti et al. described that oxytocin administration (intraperitoneally or intracerebroventricularly) reduced food intake in rats, due to both a reduced meal size and an increased latency to feeding (9). Oxytocin is now well recognized as an anorexigenic neuropeptide, which effects are mediated by reducing gastric emptying and GI transit, as well as by suppressing the feeding reward circuit (4, 10). Furthermore, oxytocin and OXTR knockout mice were shown to develop late-onset obesity (11, 12), without alterations in food intake, pointing to the fact that oxytocin is controlling metabolic homeostasis, not only via an effect on food intake but also by modulating energy expenditure (11, 13) [for review, see Ref. (4)]. In keeping with the roles of oxytocin described in rodents (see below), patients with Prader–Willi syndrome (whose main characteristics are cognitive disabilities, chronic food craving, and morbid obesity) seem to present a deficit in the oxytocin producing neurons of the PVN (14), probably due to a loss of function or deletion of *SIM1*, a transcription factor controlling the expression of the *OXT* gene (15, 16).

### Summary

Oxytocin can be considered as an anorexigenic peptide. Lack of oxytocin leads to the development of obesity in rodents, as well as in humans. This is partly independent of its effect on food intake, suggesting an additional role of oxytocin in the regulation of metabolic homeostasis.

## 2 Central Oxytocin Circuits and Interaction with Leptin in the Control of Food Intake

Different oxytocin neuron populations may fulfill different functions. Their interactions with leptin also appear to be specific for some distinct neurons. Close examination of the literature in the field allows to unravel the importance of precise leptin–oxytocin circuits in the regulation of food intake.

### 2.1 PVN to NTS oxytocin circuit

**Figure 1:**
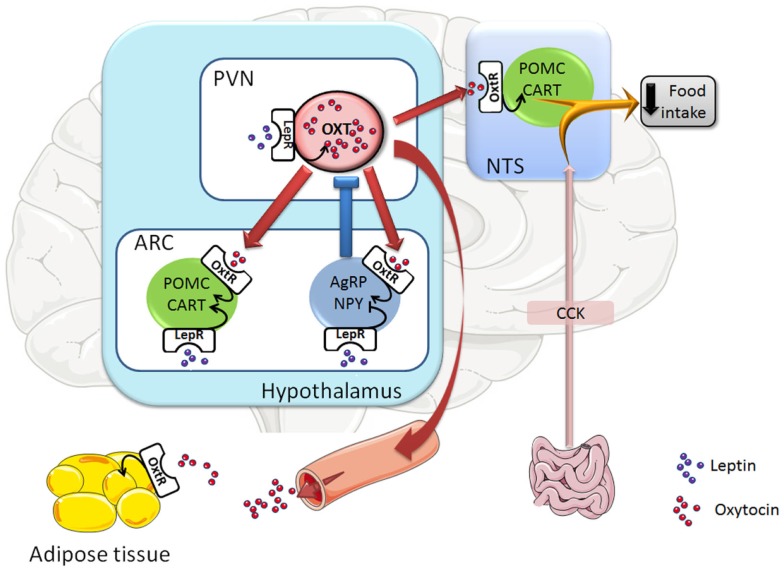
**Representative scheme of the oxytocin neuronal circuits controlling food intake**. Leptin, through the leptin receptor (LepR), activates oxytocin (Oxt) neurons in the hypothalamic paraventricular nucleus (PVN) and POMC/CART neurons in the hypothalamic arcuate nucleus (ARC), whereas it inhibits AgRP/NPY neurons in the ARC. Once oxytocin neurons are activated, they are innervating and activating POMC/CART neurons in the hypothalamic ARC nucleus and the hindbrain NTS nucleus (which is receiving also innervation from the gut), leading to a decrease in food intake. Moreover, activation of oxytocin neurons also triggers a negative feedback loop as PVN oxytocin neurons innervate AgRP/NPY neurons in the ARC nucleus, which inhibit oxytocin neurons in the PVN. At last, oxytocin neurons innervate the posterior pituitary and their activation leads to the secretion of oxytocin into the circulation, a way by which oxytocin can reach its target tissues, such as white adipose tissue. Arrow-headed lines and bar-headed lines indicate activation and inhibition, respectively. Oxytocin neurons and innervations are drawn in red, POMC/CART neurons in green, AgRP/NPY neurons in blue, leptin protein in purple circles, and oxytocin protein in red circles. Abbreviations: Oxt, oxytocin; POMC, pro-opiomelanocortin; CART, cocaine- and amphetamine-regulated transcript; AgRP, agouti-related protein; NPY, neuropeptide Y; CCK, cholecystokinin; LepR, leptin receptor; OxtR, oxytocin receptor; ARC, arcuate nucleus; PVN, paraventricular nucleus; NTS, nucleus of the solitary tract.

Accumulating evidence suggests that oxytocin neurons in the PVN mediate the anorexigenic effect of leptin (Figure 1). Thus, the oxytocin synthesizing neurons in the PVN exhibit a strong expression of the leptin receptor (17). Central leptin administration was shown to activate parvocellular oxytocin neurons of the PVN, which project to the NTS (18–20), where they are known to innervate POMC/CART neurons (21, 22). The low *Oxt* gene expression observed in the PVN during fasting was recovered after peripheral leptin administration (20, 23). Additionally, the leptin effect in decreasing food intake was partially blunted in adult mice with ablation of oxytocin neurons (24) and it was prevented by the injection of an OXTR antagonist within the fourth ventricle (19) (close to the NTS). Finally, the involvement of oxytocin neurons in leptin action was substantiated in mice with deletion of *Socs3* (an inhibitor of the Jak-Stat leptin signaling pathway) in the mediobasal hypothalamus (25), which includes the PVN. Indeed, as a result of improved leptin signaling, these mice exhibited decreased food intake and body weight. Interestingly, the oxytocin content of the dorsal vagal complex (including the NTS) was increased in response to leptin administration in these mice, and the leptin-induced decrease in food intake was abolished by injection of an OXTR antagonist into the NTS (25). Collectively, these data suggest that oxytocin neurons of the PVN could mediate the leptin-activated hindbrain (NTS)-containing satiety circuit.

One of the ways by which leptin was reported to decrease food intake is by enhancing the efficiency of gut-derived anorexigenic hormones, such as cholecystokinin (CCK) (26). Thus, in line with a role of oxytocin as a mediator of leptin’s effect on food intake, it was shown that the satiety effect of CCK is partly blunted by the central injection of an OXTR antagonist or by destruction of the OXTR expressing neurons in the NTS (27–29). In contrast, other data obtained in oxytocin-deficient mice showed that oxytocin is dispensable for feeding inhibition by CCK (24, 30). A possible explanation that could reconcile both types of results (OXTR antagonist and OXTR cell ablation vs. oxytocin knockout) is that the oxytocin antagonist used (d(CH2)5[Tyr(Me)2,Thr4,Tyr-NH2(9)] OVT) (27–29) is an inverse agonist, which would not only block the oxytocin signal but also inhibit the neurons, which express the OXTR. It is also possible that, in oxytocin knockout mice, compensatory mechanisms may offset the absence of oxytocin. Further studies are, however, needed to clarify the role of oxytocin on the anorexigenic signal from gut-derived hormones.

### 2.2 PVN to ARC oxytocin circuit

In addition to PVN oxytocin neurons projecting to the NTS, other oxytocin neurons in the PVN also innervate POMC and AgRP neurons in the ARC (7). Moreover, it was reported that AgRP neurons target and inhibit oxytocin neurons (31), constituting a short feedback loop system in the hypothalamic control of food intake. It should be mentioned, however, that intra-ARC oxytocin administration decreased food intake to a lesser extent than lateral ventricle injection, suggesting that ARC activation only partially mediates the anorexigenic effect of oxytocin (7).

### 2.3 SON oxytocin neurons

With regard to the oxytocin neurons in the SON, they also display a high expression of the leptin receptor (17). However, the leptin’s effects on oxytocin in this nucleus are not clear. Thus, icv leptin administration did not modify the *Oxt* gene expression in SON neurons (32), neither did it activate OXT neurons in this nucleus, as assessed by the measurement of c-fos expression (19). In addition, leptin was shown to exert either inhibitory or stimulatory effects on the electrical activity of SON oxytocin neurons as studied in brain slices, or *in vivo* (i.v. administration in rats) (33, 34) respectively. Further studies are required to understand the role of oxytocin in the SON, although SON oxytocin neurons do not appear to be involved in the control of food intake.

### Summary

It appears that among the hypothalamic oxytocin neurons involved in the control of food intake, those projecting from the PVN to the NTS are the most important ones in mediating the leptin’s effect.

## 3 Oxytocin Treatment in Animal Models of Obesity and Diabetes: A Way to Overcome Leptin Resistance?

Leptin administration to lean mice and rats leads to decreased food intake and body weight, effects that are blunted in obese rodents (35, 36). Moreover, diet-induced obese rodents are well known to display high-circulating leptin levels, reflecting their increased fat mass. Together, these data imply that obesity is associated with a state of leptin resistance (37). Taking into account that oxytocin is located downstream of leptin, mediating part of its effects, it would be expected that oxytocin treatment would be effective in decreasing body weight in leptin-resistant DIO animals, as well as in those with leptin or with leptin receptor deficiency.

### 3.1 Diet-induced obese rodents

Recent data demonstrate that oxytocin treatment (subcutaneous or intracerebroventricular) of DIO mice, rats and rhesus monkeys was able to decrease body weight, mainly due to a decrease in fat mass, with a concomitant improvement in glucose tolerance and insulin sensitivity (38–42). Additional results obtained using a pair-feeding paradigm or the use of low oxytocin doses (which did not alter food intake) allowed concluding that at least part of the body weight-lowering effect of oxytocin was independent from changes in food intake (38, 39). It is noteworthy at that point that the OXTR is highly expressed in adipose tissue [at a similar level as in most of the classical oxytocin target tissues (43, 44)] and is upregulated in some mice models of obesity (44, 45). This could explain the occurrence of oxytocin effects on adipose tissue, as well as the difference between the effects of the oxytocin treatment in lean and obese animals. Indeed, it was observed that chronic oxytocin administration to lean mice was almost without any effect after the first day of treatment (during which a decrease in food intake and body weight was noted) (44).

### 3.2 Rodents with leptin receptor deficiency or with impaired leptin receptor signaling

As alluded to above, oxytocin administration was also successful in treating obesity in animal models of leptin deficiency or reduced leptin receptor signaling [Zucker rats (21), Koletsky rats (41), *ob/ob* mice (44), and *db/db* mice (22, 46)]. Thus, 2 weeks of subcutaneous oxytocin infusion to *ob/ob* mice decreased body weight gain, mainly due to an effect on the fat mass. This was accompanied by a decrease in food intake, which was mainly observed during the first week of the treatment. Surprisingly, glucose tolerance of oxytocin-treated *ob/ob* mice was worsened, probably due to an increased activity of the hypothalamic–pituitary–adrenal axis, which led to increased corticosterone levels and enhanced hepatic gluconeogenesis (44). Interestingly, longer oxytocin treatment (12 weeks) in *db/db* mice also induced a decrease in body weight gain with a decrease in the fat mass, without any change in food intake. In contrast to *ob/ob* animals, oxytocin-treated *db/db* mice exhibited improved glucose tolerance and insulin sensitivity (46).

### 3.3 Oxytocin treatment in other rodent animal models

Oxytocin treatment was also demonstrated to exert beneficial effects in other animal models, improving glucose metabolism in a diabetic model of pancreatic beta cell ablation by streptozotocin (47) and decreasing fat mass gain in ovarectomized mice (48).

### Summary

Oxytocin treatment is able to overcome leptin resistance or leptin deficiency as it decreases body weight and fat mass in DIO animals, as well as in rodents with leptin deficiency or reduced leptin receptor signaling. In most experimental conditions, glucose metabolism is improved by the oxytocin treatment, except during short-term experiments in leptin-deficient mice.

## 4 Adipose Tissue as the Main Target of Oxytocin Action in Obesity

Adipose tissue seems to be one of the main targets of oxytocin in obesity, as the decrease in fat mass, in response to an *in vivo* oxytocin treatment, was observed in all the different models of rodent obesity studied so far (38, 39, 44, 46). However, considering data obtained *in vitro*, *ex vivo*, or *in vivo* during acute or chronic experiments, the reported results are contradictory.

### 4.1 *In* *Vitro*

In *ex vivo* experiments with adipose tissue or in cell cultures, oxytocin was without any effect on lipid metabolism in rabbit adipose tissue (49), whereas it reportedly increased lipogenesis (50, 51) or lipolysis (38) in rat adipocytes. It was also demonstrated that oxytocin inhibits adipocyte differentiation (52), increases glucose oxidation, and stimulates pyruvate dehydrogenase activity (53).

### 4.2 *In* *Vivo*

In acute *in vivo* experiments, oxytocin administration resulted in highly variable changes in non-esterified fatty acid (NEFA) and triglyceride (TG) levels, according to the experimental conditions used (e.g., sex, timing, feeding conditions, etc.) (54). Another acute study indicated that oxytocin increased circulating NEFA levels in dehydrated, but not in normally hydrated rats (55).

In chronic *in vivo* studies, it was shown that 15 days of treatment with oxytocin enhanced lipolysis in DIO rats (38) and promoted futile cycling (increase in both lipolysis and glycerogenesis) in *ob/ob* mice (44). In lean animals, such a chronic treatment increased adipogenesis in rats (56) and was without any effect in mice (44). Interestingly, a much longer treatment (12 weeks) was reported to decrease the size of adipocytes, the mass of fat pads, as well as to induce browning of white adipose tissue (i.e., presence of brown or beige/brite adipocytes in white adipose depots) in lean and obese *db/db* mice (46).

In humans, while acute oxytocin injection produced an increase in NEFA levels a few days after delivery (57), it resulted in decreased NEFA levels in non-pregnant healthy subjects (58). Furthermore, chronic treatment of obese humans with nasal oxytocin delivery did not significantly modify the TG levels, although there was some trend toward a decrease in this parameter (47).

### 4.3 Possible explanations for the contrary results obtained in different experimental setups

As detailed above, although *in vivo* oxytocin treatment appears to consistently reduce fat mass in several models of obesity, discrepant results pertaining to oxytocin effects on glucose metabolism were observed. Concerning oxytocin effects on adipose tissue, conflicting results were reported depending on the experimental approach used (*in vitro, ex vivo, in vivo*).

One of the main reasons underlying the description of discrepant results is the use of different models, doses, and durations of the oxytocin treatments. Alternatively, as suggested by Muchmore and colleagues (59), the contradictory data could arise from the existence of low- and high-affinity OXTRs with opposite effects in response to their activation. Thus, low-oxytocin levels would exert insulin-like activities through interaction with high-affinity OXTRs, while at high concentrations, oxytocin would inhibit glycogen deposition (likely through an activation of glycogenolysis) and induce lipolysis, through its action on the low-affinity receptors (59). It should be mentioned at that point that, although the hypothesis of dose-dependent varying affinity of oxytocin for its receptor is interesting, it was postulated more than 30 years ago using an *in vitro* approach and was not validated further since then.

Another important variable, which could influence the *in vivo* oxytocin effects, is the type of diet consumed. Indeed, by modulating the membrane composition (60), it is conceivable that the diet may modulate the affinity of oxytocin for its receptor. This was actually demonstrated in the presence of divalent cations (such as magnesium) or cholesterol (5) and could occur when animals are fed a high-fat diet to induce obesity, glucose intolerance, and insulin resistance.

Differences in the expression of the OXTR (44, 45), as well as in oxytocinase activity in adipose tissue (61) could also modulate the type of oxytocin effects on glucose or lipid metabolism.

At last, it has been demonstrated that oxytocin neurons innervate white adipose tissue through the sympathetic nervous system (62) and oxytocin knockout mice display a decreased sympathetic tone (12). Altogether, oxytocin may therefore influence adipose tissue metabolism both by direct and indirect action on this tissue via the sympathetic nervous system. Therefore, the possible different sympathetic tone in the various models used could potentially also explain some of the divergent results obtained.

### Summary

Although the data in non-obese animals and in *in vitro* experiments bring about some contradictory results, it is clear that oxytocin treatment in obese animals decreases the fat mass. In most instances, this is accompanied by improvements in glucose metabolism. The mechanisms underlying these beneficial effects remain to be determined.

## 5 Oxytocin Treatment in Obese Patients

Conflicting literature exists regarding the fact that circulating oxytocin levels are increased (63), unchanged (64), or decreased in obese (65) compared to lean human subjects. Moreover, highly variable results were reported using different types of measurements (66–68). This is an important issue as it leads to different conclusions about the activity of the oxytocin system in human obesity. This should prompt us to urgently define a standard and reliable method allowing drawing relevant conclusions on that topic.

Independently from this issue and taking into consideration that part of the leptin actions seems to be mediated by oxytocin (19), that chronic oxytocin treatment exerts promising effects on obesity and diabetes in various rodent models, as described above, it was hypothesized that oxytocin treatment could bypass the well-described leptin resistance, present not only in animals but also in obese human subjects (69).

As of now, two studies have been performed to determine the effects of oxytocin on food intake, body weight, and metabolic function in lean (70) and obese (47) volunteers. In lean healthy men (70), a single dose of 24 IU of intranasal oxytocin administration was shown to inhibit the reward – but not the hunger-driven eating, attenuating basal and postprandial levels of adrenocorticotropic hormone and of cortisol, and decreasing the postprandrial rise in plasma glucose, without modifying energy expenditure. Most interestingly, a clinical trial was performed by Zhang et al. (47), whereby intranasal oxytocin was administered to obese patients (BMI 30–36) at a dose of 24 IU, four times a day during 8 weeks. This treatment led to a significant and constant decrease in body weight (almost 9 kg) with a concomitant decrease in total- and LDL-cholesterol. It is noteworthy that this clinical trial was performed in male and female volunteers (pregnant and lactating females excluded) and that no adverse effect was described, reinforcing the fact that this treatment seems to be safe and effective in both sexes.

### Summary

The beneficial effects of oxytocin treatment on body weight homeostasis obtained in animal models of obesity seem to apply to human obesity as well.

## 6 Oxytocin and Aging

Few data are available as yet on the oxytocin system during aging. Studies reported no change in hypothalamic oxytocin content in old rats (71), total number of oxytocin neurons in old human subjects (72), or plasma oxytocin levels in aged rats or humans (73–75). This is in contrast with very recent data demonstrating a decrease in oxytocin circulating levels in aged mice (76), pointing to possible species differences. Moreover, it has been proposed that PVN oxytocin secretion is increased in aged rats (73), which would fit with the decreased food intake that accompanies aging (77).

## 7 Future Perspectives

In view of the small cohort of the clinical trial mentioned above, in which oxytocin was administered to obese patients (9 subjects in the oxytocin-treated group and 11 subjects in the placebo-treated one) (47), larger multicenter clinical trials should be performed, in order to confirm the results and extent the conclusions. Moreover, these clinical trials should include not only obese but also type 2 diabetic patients, as the study by Zhang and collaborators (47) pointed to a tendency toward improvements in postprandial glucose and insulin levels in the oxytocin-treated group of obese subjects. This would also fit the observations that rodent models of type 2 diabetes (DIO rats and mice) exhibited improvements in glucose intolerance and insulin resistance in response to oxytocin treatment. Future studies should also shed some light on the molecular mechanisms by which oxytocin treatment can exert its beneficial effects.

## Conflict of Interest Statement

Françoise Rohner-Jeanrenaud has a patent application (PCT/IB2011/052156) covering novel therapeutic uses of oxytocin. Jordi Altirriba and Anne-Laure Poher have no conflict of interest to declare.
